# Closing the THz gap with Dirac semimetals

**DOI:** 10.1038/s41377-022-00812-w

**Published:** 2022-05-06

**Authors:** Carlo Rizza, Alessandro Molle

**Affiliations:** 1 University of L’Aquila, Department of Physical and Chemical Sciences, I-67100 L’Aquila, Italy; 2 Consiglio Nazionale delle Ricerche (CNR), Istituto per la Microelettronica e Microsistemi (IMM), unit of Agrate Brianza, via C. Olivetti 2, 20864 Agrate Brianza, Italy

**Keywords:** Electronics, photonics and device physics, Optical materials and structures

## Abstract

High-performance THz photodetection is unprecedentedly accessed by integrating a topological Dirac (Weyl) semimetal in a carefully designed antenna at deep-subwavelength scales.

In recent years, there has been a surge of interest in the THz spectral regime (from 300 Hz to 30 THz) since efficient electronic and photonic materials are traditionally not available in this frequency range. Conventional electronic materials are too slowly responsive for THz optoelectronics, and mainstream optoelectronic and photonic devices (lasers, photodetectors, modulators, etc.) operating in the visible and infrared ranges cannot be trivially recast to the THz band owing to intrinsic physical constraints^1^. For example, III–V or group IV semiconductors, widely exploited in several optical devices, exhibit an energy bandgap out of the THz spectral range, and this specific strongly hampers the devising of active THz optoelectronic components.

To overcome these limitations, research efforts are currently focused on novel THz materials with unusual and advantageous properties. THz metamaterials, designer-made composite materials showing a resonant response at the desired THz frequency, provide a suitable platform to manipulate THz radiation^2,3^. However, despite the THz responsivity, metamaterials (generally studied for their effective, spatially averaged, properties) exhibit undesired deep-subwavelength spatial modulations^4^, which can be detrimental for miniaturized on-chip optoelectronic devices. An alternate fashion of technology-amenable THz materials was inspired by graphene^5^, closely followed up by three-dimensional (3D) topological insulators^6–10^, where linearly dispersing fermions can be excited by low-energy photons. Recently, a new frontier of topological materials has emerged. Especially, topological Dirac (Weyl) semimetals have attracted a good deal of attention since they exhibit topologically protected crossing points, termed Dirac (Weyl) nodes, between four-fold degenerate (two-fold non-degenerate) linearly dispersing energy bands in their 3D electronic structure^11^. Topological semimetals offer unprecedented perspectives to tune the light-matter interaction at extremely low photon energy (even down to the THz regime)^12,13^. In this context, a subclass of van der Waals solids consisting of transition-metal dichalcogenides TMX_2_ (TM = Mo, W, Pd, Pt; X = Se, Te), plays an outstanding role since they may assume allotropic phases hosting Lorentz-violating (type-II) topological Dirac or Weyl fermions in bulk crystal^14^ and potentially in the form of thin-films on substrates^15^. In these materials, the Dirac (Weyl) nodes are no longer pinned to the high-symmetry points of the crystal lattice, and they emerge at the boundary between electron and hole pockets arising from band intersections with the Fermi surface.

In ref. ^16^, Wang et al. set a milestone in THz technology by proposing the integration of the type-II Dirac semimetal PtSe_2_ in a high-performance THz photodetector, realizing a suitable nanometric antenna layout (see Fig. 1 as a general scheme of device operation). PtSe_2_ exhibits solid advantages as THz material. PtSe_2_ is stable against environmental degradation in the month timescale, thus ensuring the endurance of the topological character after integration into a technology platform. Its topological type-II Dirac semimetal character has been well-established by angle-resolved band structure investigations of tilted Dirac cones with broken Lorentz symmetry^17,18^. More importantly, it bears a broadband electromagnetic absorption at the THz frequencies, high mobility (1800 cm^2^/V s), and high anisotropic response. On the other hand, as a semimetal, PtSe_2_ can suffer from limited photo-electric conversion within a deep-subwavelength area and bias-induced dark current leakage. To overcome these latter drawbacks, the Authors propose an asymmetric metallization confining the optically active nanogap slit in the sub-skin-depth regime (*λ*/10^4^) within the antenna device architecture. Breaking the in-plane symmetry of the electrode layout effectively results in an enhanced funneling of low-energy photons and photo-induced conduction at zero bias. The considered PtSe_2_-based photodetector exhibit a colossal photo-response, viz., light absorption in the THz range (from 0.1 to 0.3 THz) with responsivity exceeding 0.2 A/W. In addition, noise-equivalent power (NEP) less than 38 pW/Hz, and temporal response of the order of 1 μs are achieved by coupling the PTSe_2_ with graphene, so as to alleviate the energy barrier at the contact. These figures of merit hold promise for PtSe_2_ as outstanding THz material and pave the way for integrating other topological materials in miniaturized on-chip optoelectronic devices. Taking the PtSe_2_ antenna as a prototypical device, new perspectives for devising ultra-efficient low-energy optoelectronic components can thus be one step closer to the realization stage and to the deployment of efficient and ready-to-use THz products that are advantageous for biomedical, remote sensing, and security applications.

**Fig. 1:**
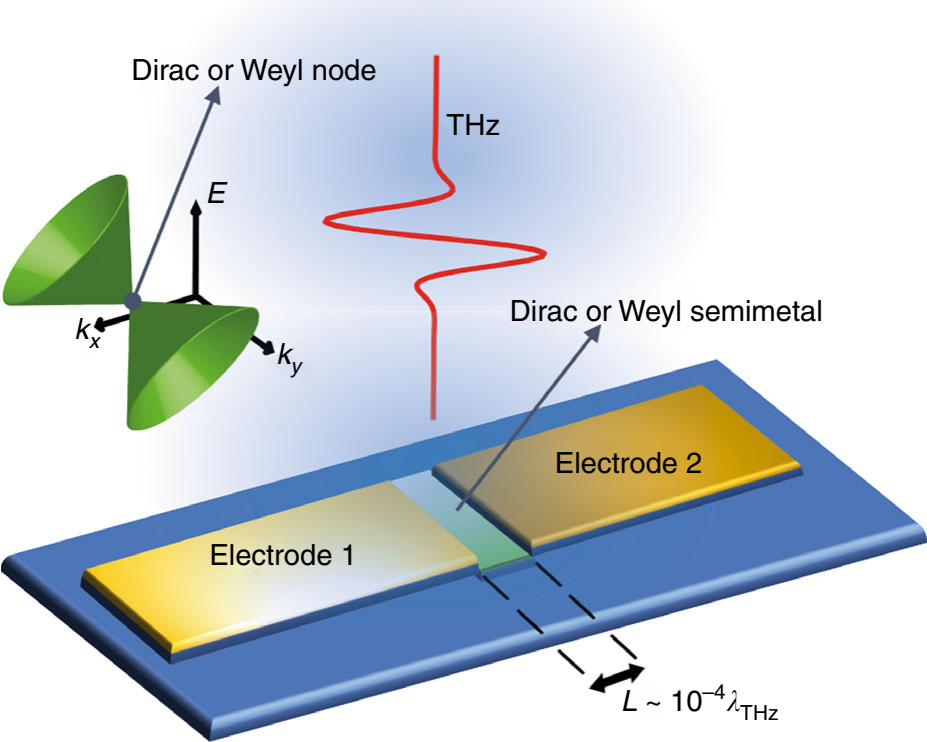
Schematic of the in-plane asymmetric device enabling an efficient low-energy photodetection. The THz near-field is concentrated into the nanogap of thickness *L*, where the type-II Dirac or Weyl semimetal lies
